# New Anti-Inflammatory Aromatic Components from *Antrodia camphorata*

**DOI:** 10.3390/ijms14034629

**Published:** 2013-02-26

**Authors:** Yu-Chang Chen, His-Lin Chiu, Che-Yi Chao, Wen-Hsin Lin, Louis Kuoping Chao, Guan-Jhong Huang, Yueh-Hsiung Kuo

**Affiliations:** 1 Department of Chinese Pharmaceutical Sciences and Chinese Medicine Resources, College of Pharmacy, China Medical University, Taichung 404, Taiwan; E-Mails: yuchang@mail.cmu.edu.tw (Y.-C.C.); f91223033@ntu.edu.tw (H.-L.C.); 2 Department of Health and Nutrition Biotechnology, College of Health Science, Asia University, Taichung 412, Taiwan; E-Mail: cychao@asia,edu.tw; 3 School of Pharmacy, College of Pharmacy, China Medical University, Taichung 404, Taiwan; E-Mail: wslin@mail.cmu.edu.tw; 4 Department of Cosmeceutics, College of Pharmacy, China Medical University, Taichung 404, Taiwan; E-Mail: kuoping@mail.cmu.edu.tw; 5 Tsuzuki Institute for Traditional Medicine, China Medical University, Taichung 404, Taiwan

**Keywords:** *Antrodia camphorata*, polyporaceae, benzenoid, anti-inflammatory

## Abstract

Three new benzenoids, 3-isopropenyl-2-methoxy-6-methyl-4,5-methylenedioxyphenol (**1**), 2-hydroxy-4,4′-dimethoxy-3,3′-dimethyl-5,6,5′,6′-bimethylenedioxybiphenyl (**2**), 4,4′-dihydroxy-3,3′-dimethoxy-2,2′-dimethyl-5,6,5′,6′-bimethylenedioxybiphenyl (**3**), together with two known benzenoids, 2,3,6-trimethoxy-5-methylphenol (**4**) and 2,3-methylenedioxy-4-methoxy-5-methylphenol (**5**), were isolated from *Antrodia camphorata*. Our results support that compounds **1**–**5** potently inhibited LPS (lipopolysaccharide)-induced nitric oxide (NO) production in a dose-dependent manner. The IC_50_ values of compounds **1**, **3** and **5** were 1.8 ± 0.2, 18.8 ± 0.6 and 0.8 ± 0.3 μg/mL, respectively.

## 1. Introduction

**Figure 1:**
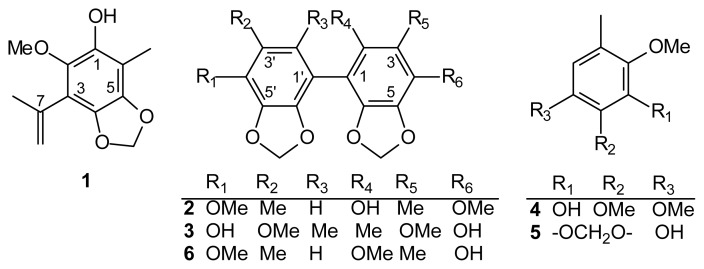
The chemical structures of compounds **1**–**5**.

*Antrodia camphorata* Wu, Ryvarden and Chang (synonym: *Ganoderma camphoratum*, *Antrodia cinnamomea*, *Taiwanofungus camphoratus*) (Polyporaceae) is a parasitic fungus on the inner wall of the heartwood of *Cinnamomun kanehiria* Hay (Lauraceae). The fruiting bodies of *A. camphorata* are called “chang-chih” or “niu-chang-chih” in Taiwan. Traditionally, the fungus has been used for the treatment of food and drug intoxication, diarrhea, abdominal pain, hypertension and liver cancer [1]. The components of this fungus have shown activities of anti-inflammation [2–12], immune-modulation [13], anti-*Helicobacter pylori*[14], neuroprotection from Aβ damage [15], anti-hepatitis-B virus [16,17] and anticancer [18–31]. Here, we present the result of chemical studies from a mixture of the fruiting body and mycelia of wood cultures of *A. camphorata* and three new benzenoids, 3-isopropenyl-2-methoxy-6-methyl-4,5-methylenedioxyphenol (**1**), 2-hydroxy-4,4′- dimethoxy-3,3′-dimethyl-5,6,5′,6′-bimethylenedioxybiphenyl (**2**), 4,4′-dihydroxy-3,3′-dimethoxy-2,2′- dimethyl-5,6,5′,6′-bimethylenedioxybiphenyl (**3**) together with two known benzenoids, 2,3,6-trimethoxy-5-methylphenol (**4**) and 2,3-methylenedioxy-4-methoxy-5-methylphenol (**5**) (Figure 1), which were isolated and elucidated.

## 2. Results and Discussion

### 2.1. Isolation and Structure Elucidation

Extensive chromatographic purification of the EtOAc-soluble fraction (Fr. A) of the MeOH extract of *A. camphorata* afforded compounds **1**–**5**.

**Figure 2:**
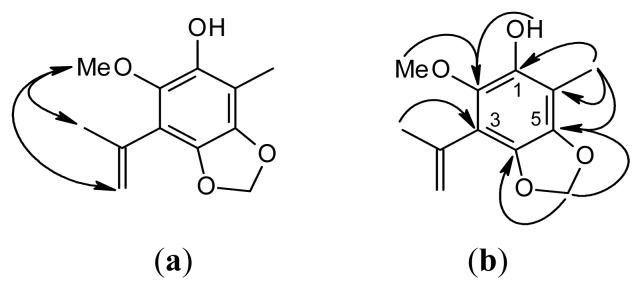
Nuclear Overhauser Effect Spectroscopy (NOESY) contacts (**a**) and key Heteronuclear Multiple Bond Correlation (HMBC) connectivities (**b**) of compound **1**.

Compound **1** was isolated as colorless oil. Its molecular formula, C_12_H_14_O_4_, was determined by High Resolution Fast Atom Bombardment Mass Spectrometry (HR-FABMS) ([M + 1]^+^, *m*/*z* 223.0963). The infrared (IR) spectral data showed the presence of the hydroxyl group (3440 cm^−1^) and the benzene ring (1618, 1510 cm^−1^). The ^1^H- and ^13^C-nuclear magnetic resonance (NMR) spectra (Table 1) of **1** showed the Heteronuclear Multiple-Quantum (HMQC) correlation, as follows: a OCH_2_O moiety [δ_H_ 5.93 (s), δ_C_ 101.7], a Me group [δ_H_ 2.27 (s), δ_C_ 13.3] and a MeO group [δ_H_ 3.94 (s), δ_C_ 60.4] on the phenol. The presence of an isopropenyl group was revealed by a Me group [δ_H_ 1.99 (s), δ_C_ 23.5], two olefinic protons of CH_2_ [δ_H_ 5.24 (br s), 5.36 (br s), δ_C_ 121.0] and a quaternary C-atom [δ_C_ 127.2 (C-7)]. On the basis of HMBC (Figure 2), cross-peaks [δ_H_ 5.93 (OCH_2_O) coupled to δ_C_ 133.0 (C-5) and 136.0 (C-4); δ_H_ 2.27 (Me) correlated to δ_C_ 110.0 (C-6), 132.0 (C-1) and 133.0 (C-5); δ_H_ 4.64 (OH) coupled to δ_C_ 132.0 (C-1); δ_H_ 3.94 (MeO) coupled to δ_C_ 138.5 (C-2); δ_H_ 1.99 (Me-7) coupled to δ_C_ 97.1 (C-3)] and a combination with the Nuclear Overhauser Effect Spectroscopy (NOESY) experiment (Figure 2) [MeO (δ_H_ 3.94) correlation with isopropenyl group (δ_H_ 1.99, 5.24, 5.36)] corroborated the locations of the functional groups on the benzene ring. On the basis of the ^1^H- and ^13^C-NMR (Table 1), NOESY (Figure 2), Distortionless Enhancement by Polarization Transfer (DEPT), HMQC and Heteronuclear Multiple Bond Correlation (HMBC) (Figure 2) experiments, **1** was characterized as 3-isopropenyl-2-methoxy-6-methyl-4,5-methylenedioxyphenol.

**Figure 3:**
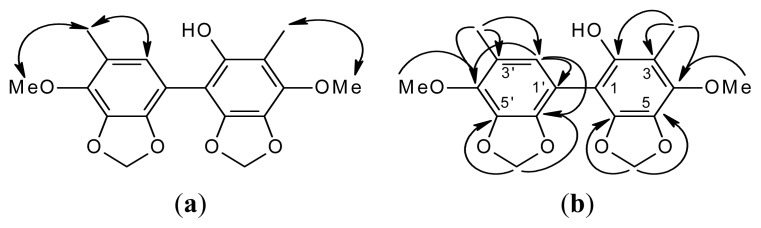
NOESY contacts (**a**) and key HMBC connectivities (**b**) of compound **2**.

Compound **2** was isolated as an amorphous solid. Its molecular formula, C_18_H_18_O_7_, was determined by HR-FABMS ([M + 1]^+^, *m*/*z* 347.1128). The presence of phenolic moiety was revealed by IR spectral data (3481, 1615, 1512 cm^−1^). The above data combined with the NMR data (Table 1) revealed **2** to be a biphenyl compound. The ^1^H-NMR spectrum (Table 1) of **2** showed two OCH_2_O groups [δ_H_ 5.96 (s, 5-OCH_2_O-6), 5.98 (s, 5′-OCH_2_O-6′)], two MeO groups [δ_H_ 3.87 (s, MeO-4′), 3.88 (s, MeO-4)], two Me groups [δ_H_ 1.97 (s, Me-3), 2.03 (s, Me-3′)] and a single aromatic proton [δ_H_ 5.94 (s, H-2′)]. The ^13^C-NMR (Table 1) and DEPT spectra showed that **2** had a total of 18 C-atoms, accounting for two Me [δ_C_ 9.42 (Me-3), 15.8 (Me-3′)], two MeO [δ_C_ 59.7 (MeO-4′), 60.1 (MeO-4)], two OCH_2_O [δ_C_ 101.7 (5′-OCH_2_O-6′), 101.8 (5-OCH_2_O-6)], one aromatic CH [δ_C_ 109.5 (C-2′)] and 11 aromatic quaternary C-atoms [δ_C_ 116.8 (C-3), 124.1 (C-3′), 129.3 (C-1), 133.4 (C-6), 134.4 (C-6′), 135.0 (C-4), 135.6 (C-2), 136.0 (C-5), 136.1 (C-1′), 137.4 (C-4′), 138.6 (C-5′)]. These data also indicated a biphenyl skeleton. Assignment of chemical shifts of all protonated C-atoms and their associated H-atoms in the molecule can be finished according to HMQC data. On the basis of HMBC (Figure 3), cross-peaks of MeO-4 with C-4, of MeO-4′ with C-4′, of Me-3 with C-2, C-3 and C-4, of Me-3′ with C-2′, C-3′ and C-4′, of 5-OCH_2_O-6 with C-5 and C-6, of 5′-OCH_2_O-6′ with C-5′ and C-6′ and of H-2′ with C-1′, C-4′ and C-6′, the remaining C-atoms of the aromatic ring, C-1, were assigned. The NOESY experiment (Figure 2) showing Me-3 correlated with MeO-4 and Me-3′ correlated with H-2′ and MeO-4′ further supported the substitution pattern. According the above evidence, compound **2** can be assigned as structures **2** or **6** (Figure 1). The statistical calculation from a text book [32] suggested that the difference of ^13^C chemical shift between C-2 and C-4 is slight for structure **2** and larger for structure **6**. Therefore, we assigned the compound **2,** as structure **2** is more reasonable, and structure **6** will be excluded. On the basis of the ^1^H- and ^13^C-NMR (Table 1), NOESY (Figure 2), DEPT, HMQC and HMBC (Figure 3) experiments and comparison of ^13^C-NMR values between C-2 and C-4, compound **2** was characterized as 2-hydroxy-4,4′-dimethoxy-3,3′-dimethyl-5,6,5′,6′-bimethylenedioxybiphenyl (Figure 3).

**Figure 4:**
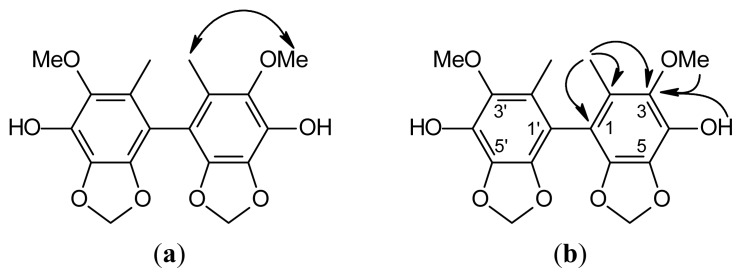
NOESY contacts (**a**) and key HMBC connectivities (**b**) of compound **3**.

Compound **3** was isolated as an amorphous solid. Its molecular formula, C_18_H_18_O_8_, was determined by HR-FABMS ([M + 1]^+^, *m*/*z* 363.1076). The presence of phenolic moiety was revealed by IR spectral data (3421, 1605, 1508 cm^−1^). According to the molecular formula, IR spectrum combined with nine ^13^C-NMR signals indicated that compound **3** is a symmetrical biphenolic derivative. These data with the NMR data (Table 1) suggest a biphenyl compound. The ^1^H-NMR spectrum (Table 1) of **3** showed a OCH_2_O group [δ_H_ 5.99 (s, 5-OCH_2_O-6)], a MeO group [δ_H_ 3.88 (s, MeO-3)], a Me group [δ_H_ 1.82 (s, Me-2)] and a hydroxy group [δ_H_ 4.56 (s, HO-4)]. The ^13^C-NMR (Table 1) and DEPT spectra of **3** showed nine signals, accounting for a Me [δ_C_ 12.7 (Me-2)], a MeO [δ_C_ 60.1 (MeO-3)], a OCH_2_O [δ_C_ 101.7 (5-OCH_2_O-6)] and six aromatic quaternary C-atoms (δ_C_ 114.6 (C-2), 123.7 (C-1), 133.2 (C-4), 133.3 (C-6), 136.0 (C-3), 138.9 (C-5). Because HR-FABMS showed that the molecular formula is C_18_H_18_O_8_, **3** was suggested to be a symmetrical biphenolic compound. The HMBC data (Figure 4) showed that the H-atom signal of Me-2 correlated to the C-atom signals of C-1, C-2 and C-3 and the H-atom signals of MeO-3 and HO-4 correlated to the C-atom signal of C-3, suggesting that OCH_2_O group was positioned at C-5 and C-6. On the basis of the ^1^H- and ^13^C-NMR (Table 1), NOESY (Figure 4), DEPT, HMQC and HMBC (Figure 4) experiments, **3** was characterized as 4,4′-dihydroxy-3,3′-dimethoxy-2,2′-dimethyl-5,6,5′,6′-bimethylenedioxybiphenyl (Figure 4). The known isolates, 2,3,6-trimethoxy-5-methylphenol (**4**) [33] and 2,3-methylenedioxy-4-methoxy- 5-methylphenol (**5**) [33], were readily identified by comparison of physical and spectroscopic data (UV, IR, ^1^H NMR and mass spectrometry data) with values found in the literature.

### 2.2. Anti-Inflammatory Activities

Compounds **1**–**5** were evaluated for anti-inflammatory activities and exhibited the potential inhibition against LPS (lipopolysaccharide)-induced NO in a dose-dependent manner (Table 2). The IC_50_ values of compounds **1**, **3** and **5** were 1.8 ± 0.2, 18.8 ± 0.6 and 0.8 ± 0.3 μg/mL, respectively (Table 2).

Compounds **5** is very potent (IC_50_ = 0.8) for the inhibition of NO production. We will study the anti-inflammatory activities of compound **5** further.

## 3. Experimental Section

### 3.1. General

Column chromatography (CC): silica gel 60 (Merck 70–230 mesh, 230–400 mesh, ASTM). Prep. HPLC: (LDC Analytical-III system; column: LiChrosorb Si 60, 7 μm, 250 × 10 mm). UV: Hitachi S-3200 spectrometer; λ_max_ (log ɛ) in nm. IR spectra: Perkin-Elmer 983G spectrophotometer; ν in cm^−1. 1^H-, ^13^C- and 2D-NMR spectra: Bruker DMX-500 spectrometer; δ in ppm rel. to TMS, *J* in Hz. HR-FABMS: *JEOL SX-102A* spectrometer; *m*/*z*.

### 3.2. Plant Material

The solid cultural fruiting bodies of *A. camphorata* were identified and provided by Po-Zone Biotechnology Development, Taipei, Taiwan. A voucher specimen was deposited at Po-Zone Biotechnology Development Co. Ltd.

### 3.3. Extraction and Isolation

The fruiting bodies of wood culture *A. camphorata* (500 g) were extracted with MeOH (4 L) by maceration at room temperature (7 days × 3). After removal of MeOH under vacuum, the extract was partitioned into EtOAc (Fr. A, 113 g), *n*-BuOH (Fr. B, 15 g) and H_2_O-soluble (Fr. C, 27 g) fractions. The EtOAc fraction (Fr. A, 113 g) was subjected to CC (10 × 70 cm, silica gel, 230–400 mesh) using n-hexane, EtOAc and MeOH of increasing polarity as eluent to obtain 11 fractions: Frs. A1–A18. Fr. A4 (11 g, *n*-hexane/EtOAc 8:2) was subjected to HPLC (CH_2_Cl_2_/EtOAc 9:1) to yield **4** (11.9 mg) and **5** (73.2 mg). Fr. A5 (30 g, *n*-hexane/EtOAc 7:3) was subjected to HPLC (CH_2_Cl_2_/EtOAc 9:1) to yield **1** (4.2 mg), **2** (7.3 mg) and **3** (5.1 mg).

### 3.4. 3-Isopropenyl-2-methoxy-6-methyl-4,5-methylenedioxyphenol (**1**)

Colorless oil. UV (MeOH): 280 (3.92). IR (neat): 3440, 1618, 1510, 1470, 1230, 1061, 1026. ^1^H- and ^13^C-NMR: see Table 1. HR-FABMS *m*/*z*: 223.0963 [M + 1]^+^ (C_12_H_15_O_4_^+^, calc. 223.0970).

### 3.5. 2-Hydroxy-4,4′-dimethoxy-3,3′-dimethyl-5,6,5′,6′-bimethylenedioxybiphenyl (**2**)

Amorphous solid. UV (MeOH): 276 (3.86). IR (KBr): 3481, 1615, 1512, 1468, 1240, 1155. ^1^H- and ^13^C-NMR: see Table 1. HR-FABMS *m*/*z*: 347.1128 [M + 1]^+^ (C_18_H_19_O_7_^+^, calc. 347.1131).

### 3.6. 4,4′-Dihydroxy-3,3′-dimethoxy-2,2′-dimethyl-5,6,5′,6′-bimethylenedioxybiphenyl (**3**)

Amorphous solid. UV (MeOH): 270 (3.74). IR (KBr): 3421, 1605, 1508, 1472, 1233, 1130, 1089. ^1^H- and ^13^C-NMR: see Table 1. HR-FABMS *m*/*z*: 363.1076 [M + 1]^+^ (C_18_H_19_O_8_^+^, calc. 363.1080).

### 3.7. Chemicals

LPS (endotoxin from *Escherichia coli*, serotype 0127:B8), Carr (type IV), indomethacin, MTT (3-[4,5-dimethylthiazol-2-yl]-2,5-diphenyltetrazolium bromide) and other chemicals were purchased from Sigma-Aldrich Chemical Co. (St. Louis, MO, USA).

### 3.8. Cell Culture

A murine macrophage cell line, RAW264.7 (BCRC No. 60001), was purchased from the Bioresources Collection and Research Center (BCRC, Hsinchu, Taiwan) of the Food Industry Research and Development Institute (Hsinchu, Taiwan). Cells were cultured in plastic dishes containing Dulbecco’s Modified Eagle Medium (DMEM, Sigma, St. Louis, MO, USA) supplemented with 10% fetal bovine serum (FBS, Sigma) in a CO_2_ incubator (5% CO_2_ in air) at 37 °C and subcultured every 3 days at a dilution of 1:5 using 0.05% trypsin-0.02% EDTA in Ca^2+^-, Mg^2+^-free phosphate-buffered saline (DPBS).

### 3.9. Cell Viability

Cells (2 × 10^5^) were cultured in 96-well plate containing DMEM supplemented with 10% FBS for 1 day to become nearly confluent. Then, cells were cultured with compounds **1**–**5** in the presence of 100 ng/mL LPS (lipopolysaccharide) (*Eschericha coli* 026:B6; Sigma-Aldrich, St. Louis, Mo) for 24 h. After that, the cells were washed twice with DPBS and incubated with 100 μL of 0.5 mg/mL MTT for 2 h at 37 °C testing for cell viability. The medium was then discarded, and 100 μL dimethyl sulfoxide (DMSO) was added. After 30-min incubation, absorbance at 570 nm was read using a microplate reader (Molecular Devices, Sunnyvale, CA, USA).

### 3.10. Measurement of Nitric Oxide/Nitrite

NO production was indirectly assessed by measuring the nitrite levels in the cultured media and serum determined by a colorimetric method based on the Griess reaction. The cells were incubated with different concentrations of samples in the presence of LPS (100 ng/mL) at 37 °C for 24 h. Then, cells were dispensed into 96-well plates, and 100 μL of each supernatant was mixed with the same volume of Griess reagent (1% sulfanilamide, 0.1% naphthyl ethylenediamine dihydrochloride and 5% phosphoric acid) and incubated at room temperature for 10 min; the absorbance was measured at 540 nm with a Micro-Reader (Molecular Devices).

### 3.11. Statistical Analysis

IC_50_ values were estimated using a non-linear regression algorithm (Sigma Plot 8.0; SPSS Inc., Chicago, IL, USA). Statistical evaluation was carried out by one-way analysis of variance (ANOVA, followed by Scheffe’s multiple range tests).

## 4. Conclusions

3-Isopropenyl-2-methoxy-6-methyl-4,5-methylenedioxyphenol (**1**), 2-hydroxy-4,4′-dimethoxy-3,3′- dimethyl-5,6,5′,6′-bimethylenedioxybiphenyl (**2**) and 4,4′-dihydroxy-3,3′-dimethoxy-2,2′-dimethyl- 5,6,5′,6′-bimethylenedioxybiphenyl (**3**) are new compounds from *A. camphorata*. Compounds **1**, **3** and **5** displayed a significant concentration-dependent inhibition of NO production with IC_50_ values 1.8 ± 0.2, 18.8 ± 0.6 and 0.8 ± 0.3 μg/mL, respectively.

## Figures and Tables

**Table 1:** ^1^H- and ^13^C-nuclear magnetic resonance (NMR) data (CDCl_3_, 500 and 125 MHz, resp.) of Compounds **1**–**3**. Chemical shifts δ in ppm rel. to TMS, *J* in Hz. For atom numbering, see the Formulae.

Position	1		2		3	
		
	δ_H_	δ_C_	δ_H_	δ_C_	δ_H_	δ_C_
1	–	132.0	–	129.3	–	123.7
2	–	138.5	–	135.6	–	114.6
3	–	97.1	–	116.8	–	136.0
4	–	136.0	–	135.0	–	133.2
5	–	133.0	–	136.0†	–	138.9‡
6	–	110.0	–	133.4†	–	133.3‡
7	–	127.2	–	–	–	–
Me-2	–	–	–	–	1.82 (s)	12.7
Me-3	–	–	1.97 (s)	9.4	–	–
Me-6	2.27 (s)	13.3	–	–	–	–
MeO-2	3.94 (s)	60.4	–	–	–	–
MeO-3	–	–	–	–	3.88 (s)	60.1
MeO-4	–	–	3.88 (s)	60.1	–	–
Me-7	1.99 (s)	23.5	–	–	–	–
CH_2_-7	5.24 (br s)	121.0	–	–	–	–
	5.36 (br s)					
4-OCH_2_O-5	5.93 (s)	101.7	–	–	–	–
5-OCH_2_O-6	–	–	5.96 (s)	101.8	5.99 (s)	101.7
1′	–	–	–	136.1	–	123.7
2′	–	–	5.94 (s)	109.5	–	114.6
3′	–	–	–	124.1	–	136.0
4′	–	–	–	137.4	–	133.2
5′	–	–	–	138.6	–	138.9
6′	–	–	–	134.4	–	133.3
Me-2′	–	–	–	–	1.82 (s)	12.7
Me-3′	–	–	2.03 (s)	15.8	–	–
MeO-3′	–	–	–	–	3.88 (s)	60.1
MeO-4′	–	–	3.87 (s)	59.7	–	–
5′-OCH_2_O-6′	–	–	5.98 (s)	101.7	5.99 (s)	101.7
OH	4.64 (s)	–	–	–	4.56 (s)	–

† exchangeable;

‡ exchangeable.

**Table 2:** Cell viability and effect of compounds **1**–**5** on LPS-induced NO production in macrophages a.

Compound	Dose (μg/mL)	Cell viability (% of control)	NO level (μM)	IC_50_ (μg/mL)
control	(−)	96.4 ± 4.3	2.5 ± 0.2	
LPS	(+)	97.0 ± 0.8	25.3 ± 3.0 ###	

**1**	0.312	92.0 ± 3.3	14.5 ± 2.2 **	1.8 ± 0.2
	0.625	91.0 ± 3.7	14.1 ± 1.5 **	
	1.25	90.4 ± 2.4	13.0 ± 1.5 ***	
	2.5	87.3 ± 2.3	12.0 ± 1.7 ***	
	5	65.5 ± 1.7	(−)	

**2**	0.312	98.0 ± 1.5	16.4 ± 2.4 **	
	0.625	96.6 ± 7.6	16.1 ± 1.3 **	
	1.25	96.6 ± 2.2	15.7 ± 2.0 **	
	2.5	92.7 ± 1.3	15.5 ± 1.9 **	
	5	71.4 ± 2.2	(−)	

**3**	3.12	95.1 ± 2.9	13.7 ± 0.1 ***	18.8 ± 0.6
	6.25	93.1 ± 2.7	13.4 ± 0.4 ***	
	12.5	93.0 ± 2.6	13.2 ± 0.1 ***	
	25	91.0 ± 7.7	12.1 ± 0.6 ***	
	50	64.8 ± 2.2	(−)	

**4**	0.312	97.0 ± 1.1	20.6 ± 1.2 *	
	0.625	96.2 ± 2.2	19.4 ± 2.0 *	
	1.25	95.0 ± 2.1	18.3 ± 0.3 **	
	2.5	94.6 ± 1.6	13.6 ± 0.6 ***	
	5	69.3 ± 2.1	(−)	

**5**	0.312	93.8 ± 2.9	15.2 ± 1.4 **	0.8 ± 0.3
	0.625	88.5 ± 1.5	12.8 ± 1.9 ***	
	1.25	85.0 ± 2.9	12.1 ± 1.6 ***	
	2.5	83.8 ± 1.9	10.4 ± 1.3 ***	
	5	82.4 ± 2.7	10.0 ± 2.2 ***	

Indomethacin	25	96.2 ± 1.1	19.2 ± 0.6 *	
	50	94.8 ± 1.3	14.3 ± 0.8 **	

a The data were presented as the mean ± SD for three different experiments performed in triplicate.

### Compared with sample of the control group.

* *p* < 0.05,

** *p* < 0.01 and

*** *p* < 0.001 were compared with the LPS-alone group.
